# The effects of β-caryophyllene on butyrate utilization and metabolism in Caco-2 cells

**DOI:** 10.1038/s41598-026-46790-6

**Published:** 2026-04-01

**Authors:** H. Scroggins, C. Kent-Dennis, J. May, D. L. Harmon, J. L. Klotz

**Affiliations:** 1 Department of Animal and Food Sciences, University of Kentucky, Lexington, KY USA; 2 USDA-ARS Forage-Animal Production Research Unit, University of Kentucky Campus, Lexington, KY 40546 USA

## Abstract

Β-caryophyllene (BCP) is a plant compound that may exert bioactive effects on intestinal epithelial cell (IEC) function, including metabolism of butyrate, which is important for regulating cellular functions. The goal of this study was to investigate the effects of BCP on butyrate utilization in IEC. Caco-2 cells were grown on hanging, semi-permeable inserts as a model for the intestinal barrier. Treatments, including vehicle control, 40 µM BCP, 2 mM butyrate, and BUT plus BCP were added to the apical side of the insert and incubated for 24 and 48 h. Transepithelial electrical resistance (TEER; ohms/cm^2^) was recorded to estimate barrier function and supernatant samples were taken from apical and basolateral sides. Butyrate and β-hydroxybutyrate (BHB) concentrations were measured by LC–MS to evaluate utilization of butyrate. Targeted gene expression analysis evaluated molecular modulation of BCP. Results suggested that the addition of BCP plus butyrate increased resistance compared to butyrate alone. The addition of BCP plus butyrate resulted in increased BHB production and greater butyrate disappearance. An upregulation of sodium-glucose transport 1 (*SLC5A1*), monocarboxylic transporters 1 and 4 (*SLC16A1 and SLC16A3),* and sodium hydrogen transporter 3 (*SLC9A3*) expression, and downregulation of 3-hydroxybutyrate dehydrogenase 1 (*BDH1)* and glucose transporter 2 (*SLC2A2*) expression were observed. These data indicate that BCP may promote the utilization of butyrate in intestinal epithelial cells and may influence barrier integrity. Overall, the results of this study support further investigation into the therapeutic potential of BCP.

**Supplementary Information:**

The online version contains supplementary material available at 10.1038/s41598-026-46790-6.

## Introduction

Β-caryophyllene (BCP) is a plant compound that may exert potentially beneficial effects on intestinal epithelial cells by the modulation of nutrient utilization. β-caryophyllene can be found in many plants, including hemp, thyme, clove, oregano, and hops^1^. BCP is a naturally occurring terpene and is “generally regarded as safe” by the Food and Drug Administration (USFDA) and the European Food Safety Authority as a flavoring agent to be used in cosmetics and food as terpenes play a role in the plants aroma^1^. β-caryophyllene is a selective agonist of the cannabinoid receptor type 2 (CB2), a component of the endocannabinoid system, expressed in peripheral tissues, including the gastrointestinal tract^2^. Previous investigations have shown that BCP and activation of CB2 can modulate nutrient utilization and homeostasis^1^. Dysfunction in pancreatic β-cells, resulting in changes in glucose and insulin levels, can be mitigated with BCP^3^. Altered lipid digestion and metabolism can result in obesity and diabetes, and BCP has been successful in enhancing the oxidation rate of fatty acids and helping to maintain lipid homeostasis in a model of differentiated C2C12 myotubes^4^.

Butyrate, a short-chain fatty acid produced by microbial fermentation, is a preferred energy substrate for both ruminal epithelial cells and intestinal enterocytes^5^. The metabolism of butyrate involves butyrate undergoing β-oxidation, where it then has two fates: can enter the TCA cycle to produce ATP, or it undergoes ketogenesis to produce β-hydroxybutyrate^6^. Butyrate has been shown to have a positive impact on tight junctions by activation of the Akt/mTOR mediated protein synthesis, and is needed to maintain the intestinal barrier integrity^7^. Research has also shown that supplementation of butyrate can affect the metabolism and health of epithelial tissues. For example, It was observed that α-amino-nitrogen utilization increased in the portal-drained viscera with butyrate supplementation, which suggests that the proliferation of ruminal epithelial tissue benefited from butyrate^8^. Studies have additionally indicated that the supplementation of sodium butyrate increased average daily gains compared to the control groups, suggesting an overall effect on production^9^.

Few studies have explored the bioactive effects of BCP in the gastrointestinal tract (GIT)^10,11^. Even fewer studies have investigated the effects of BCP on epithelial cell nutrient uptake and metabolism^4^. Investigation into the effects of BCP on GIT epithelial cells would contribute to the understanding of how this compound interacts at a cellular level and potentially alter their physiological functions. This information would provide justification of further investigation into the benefits of BCP, for both humans and animals. Therefore, the objective of this study was to investigate the effects of BCP on butyrate utilization in intestinal epithelial cells using Caco-2 cell monolayers as a model for the intestinal barrier. It was hypothesized that BCP would promote the utilization of butyrate by intestinal epithelial cells, as other research has noted enhancement of fatty acid oxidation^4^. To test this hypothesis, we conducted an initial experiment (Experiment 1) to evaluate butyrate metabolism in Caco-2 cells when exposed to BCP. I a second, independently-conducted experiment (Experiment 2), we sought to verify our initial findings with a greater sample size, and additionally investigate the effects on relevant genes of interest.

## Materials and methods

### Viability: AlamarBlue assay

Certified reference BCP (Sigma-Aldrich; Cat. CRM40483) was used for all experiments in this study. Cell viability was estimated using alamarBlue (Thermo Fisher Scientific, Waltham, MA) on cells (n = 4 plates) in 24-well plates. Each plate used was derived from an independent growth flask of cells, presenting each experimental unit, which were seeded in replicate wells. Previous work, albeit limited, in cell culture models used a wide range of BCP concentrations^4,12–14^. The cells were therefore exposed to 0, 10, 40, 80, 100 µM of BCP in a volume of 0.5 mL/well, with duplicates per treatment, per plate. Cells were exposed for 24 and 48 h, followed by the addition of the alamarBlue dye (final concentration of 10% v/v) to each well. Cells were then incubated at 37 °C for 2 h. Subsequently, triplicate, 100 µl samples of supernatant were collected from each well and transferred to a 96-well plate. Using a microplate reader, fluorescence was read at 560 excitation/590 emission nm. The following equation was used to quantify the percent change in cellular metabolic reduction:

$$= \frac{{{\text{Flourescence }}\;{\text{Intestisty }}\;{\text{at }}\;{590}\;{\mathrm{nm}}\;{\text{ of}}\;{\text{ Sample}}}}{{{\mathrm{Flourescence}}\;{\text{ Intestinity}}\;{\text{ at }}\;{590}\;{\text{nm }}\;{\mathrm{of}}\;{\text{ Control}}}}*100$$

### Cell establishment

For subsequent experiments, Caco-2 cells (ATCC, Manassas, VA, USA) were grown in minimum essential medium (Sigma-Aldrich, St. Louis, MO, USA) supplemented with 2% HEPES (Sigma), 10% fetal bovine serum (FBS; Thermo Fisher Scientific, Waltham, MA, USA), 1% antibiotic–antimycotic combination (final concentrations of 100 U/mL penicillin, 100 μg/mL streptomycin, and 0.25 μg/mL amphotericin B; Thermo Fisher), 1% nonessential amino acids (Sigma) to make minimum essential medium complete (MEMC) in T75 flasks in a humidified 37 °C incubator with 5% CO_2_. The MEMC was then changed every 3–4 days. Cells were split 1:5 when flasks were between 80 and 90% confluent. For experiments, cells were seeded onto a 12 mm hanging insert (Transwell; Corning Inc., Corning, New York, USA) with 0.4 µm pore size (n = 4 plates) at a seeding rate of 5 × 10^4^ cells per well. The cells were grown for 18 days with media changes every 2–3 days on both the apical (top) and basolateral (bottom) sides of the insert. Replacement media consisted of 0.6 mL of media on top and 1.2 mL of media on the bottom of each well. Based on the viability results in the present manuscript, and concentrations used previous studies^4,12–14^, we chose a moderate BCP concentration of 40 µM to use for the remainder of the study. The experimental design is outlined in Supplemental Fig. S1.

### Experiment 1

Three days before starting the experiment, media were replaced with MEMC + 5% FBS, to gradually acclimate the cells to low FBS and finally to 1% FBS on day −1 to minimize background interference on the mass spectrometer when analyzing the samples. On d 19, old media was removed, and 1 of 4 treatments was added to triplicate wells per plate. Each plate was seeded as an independent experimental unit. Treatments included vehicle control (VC), 40 µM BCP (BCP; Sigma, CRM40483), 2 mM butyrate (BUT; Sigma), and BUT plus BCP (BB). Final concentrations of all compounds were added to the cells in MEMC + 1% FBS, with 0.6 mL apically, and 1.2 mL MEMC + 1% FBS on the basolateral side. Cells were incubated with compounds for 24 and 48 h, and supernatant samples were taken from both the apical and basolateral side at each time point.

### TEER and LC–MS analysis

Transepithelial electrical resistance (TEER; ohms/cm^2^) readings were taken using a voltohmmeter (Millicell ERS-2; Millipore, World Precision Instruments, Sarasota, FL) at three time points (T0, T24, T48) to check the confluency on the membrane and estimate barrier integrity.

Supernatant samples from both the apical and basolateral sides of the cell layer were taken at each time point and stored at −20 °C to later be analyzed for butyrate and BHB concentrations by liquid chromatography-mass spectrometry (LCMS). A 100-µL aliquot of sample underwent protein precipitation via the addition of acetonitrile containing two isotopically labeled internal standards, butyric acid-d_7_(Cayman Chemical Company, Ann Harbor, MI) and sodium hydroxybutyrate-d_4_ (CDN Isotopes, Pointe-Claire, QC, Canada), the samples were vortexed and centrifuged. Derivatizing agents, 3-Nitrophenylhydrazine (3-NPH) (Sigma), and EDAC (MilliporeSigma, Burlington, MA) plus 7% pyridine (Sigma), were added (and samples heated) to create 3-NPH derivatives of analytes. Samples were analyzed on a UPLC-MS (Waters Acquity H-Plus UPLC with a Water Xevo TQ-S Cronos Mass Spectrometer; Waters Corp, Milford, MA), operated in negative electrospray ionization, utilizing multiple reaction monitoring (MRM) modes. Separation of analytes was obtained using a gradient program (supplemental Table S1) on a C18 column (Waters Acquity BEH C18 (2.1 mm × 150 mm × 1.7 μm)) with a mobile phase consisting of water and acetonitrile (both with 0.1% formic acid). The MS was optimized using authentic standards, and samples were quantified against linear calibration curves (ranging from 1 to 2500 µM) using quantitation ion transitions for all the analytes and their corresponding internal standards (See supplemental Table S2). Quality control consisted of blanks, duplicates, and spiked samples to assess the precision and accuracy of the analysis method. BHB concentrations were normalized per volume of supernatant.

### Experiment 2

For experiment 2, 5 plates were dedicated to 24 h and 5 plates were dedicated to 48 h in order to collect cells at each time point. Each plate was seeded as an independent experimental unit. The TEER was measured as described for Experiment 1. Treatment and exposure times for this experiment were the same as for Experiment 1. Supernatants were collected and cells were lysed in tri-reagent (Trizol; Thermo) at 24 and 48 h. Samples were run on the LC–MS for β-hydroxybutyrate and butyrate concentrations as described for Experiment 1.

### Total RNA extraction and real time qPCR

Total RNA extraction was completed with a phenol–chloroform extraction method^15^. Two additional isopropanol precipitations were performed during the extraction procedure for sample cleanup and a co-precipitant, linear acrylamide (Thermo), was added to help with visualization. Following extraction, samples were DNase-treated using a DNase kit according to the manufacturer’s instructions (Turbo DNase; Thermo). RNA integrity numbers of at least 8 were confirmed using a bioanalyzer (Agilent Technologies, Santa Clara, CA, USA). Samples were reverse-transcribed to make cDNA using reverse transcription (High-Capacity cDNA Reverse Transcription Kit Thermo) and diluted in nuclease-free water to a final concentration of 10 ng/μL. Duplicate samples with 20 ng of cDNA were analyzed using quantitative real-time PCR (RT-qPCR) using SYBR Green (Fast SYBR Green Master Mix; Thermo) in a real-time PCR system (StepOnePlus; Thermo). A melt curve was performed following amplification to confirm a single product. Reference genes and target genes of interest are listed in Table 1. Primer efficiency via serial dilution of pooled cDNA was between 90 and 110%. Target genes were normalized to the geometric mean of three stable housekeeping genes, *GAPDH*, *STX5*, and *HRPT*. Statistical analysis was performed on ∆Ct, however, data are presented as fold change.

**Table 1:** Primers used in RT-qPCR. The table includes gene name, sequence (5’-3’), amplicon size (bp), efficiency (%), and target reference sequence.

Gene ID	Gene name	Sequence (5’-3’)^1^	Amplicon size (bp)	Efficiency (%)^2^	Target RefSeq^3^
Housekeeping					
*GAPDH*	Glyceraldehyde-3-phosphate dehydrogenase	F: GGGTCATCATCTCTGCACCT R: GGAGGCATTGCTGACAATCT	101	91	NM_001034034.2
*STX5*	Syntaxin 5	F: CCATTCAGAGGATCGACGAG R: GGATGTGACCGACTGGAAGT	95	103	NM_001075444.1
*HPRT*	Hypoxanthine–guanine phosphoribosyltransferase	F:TGGACAGGACTGAACGTCTT R:AATCCAGCAGGTCAGCAAAG	114	92	NM_003164.5
Targets					
*PPARA*	Peroxisome proliferator-activated receptor alpha	F: CGGACACGCTTTCACCAG R: CCCCGCAGATTCTACATTCG	114	92	NM_005036.6
*PPARG*	Peroxisome proliferator-activated receptor gamma	F:ACAACAGACAAATCACCATTCGT R: CCACCTCTTTGCTCTGCTC	112	108	NM_138711.6
*PPARD*	Peroxisome proliferator-activated receptor delta	F: AGGAGAAAGAGGAAGTGGCA R:GGGAGAGGTCTGTGTAGCTG	96	106	NM_006238.5
*BDH1*	3-hydroxybutyrate dehydrogenase 1	F: TGCTTGATGAAGGACAAAGGC R: ACTTTCTCCACCTCTTCGCT	113	97	NM_004051.5
*SLC5A1* (SGLT1^4^)	Sodium-glucose cotransporter 1	F: CCTGACTGGGTTTGCTTTTCA R: CGGAAGATGTGGAAGGAGTC	150	96	NM_000343.4
*SLC2A2* (GLUT2^4^)	Glucose transporter 2	F: CCTGGTTCATGGTGGCTGA R: CAGAAGTCCGCAATGTACTGG	127	101	NM_000340.2
*SLC16A1* (MCT1^4^)	Monocarboxylate transporter 1	F: TTTGAAACATTGATGGACCTTGT R: CATGTCATTGAGCCGACCTAAA	123	92	NM_003051.4
*SLC16A3* (MCT4^4^)	Monocarboxylate transporter 4	F: CATCACGGGGTTGGGTTTG R: AGGGCACACAGGAAGACAG	129	102	NM_004207.4
*SLC9A2* (NHE2^4^)	Sodium-hydrogen exchanger 2	F: TGCATCTCTAAATGATTGTCGTG R: TCGGCTGTCAGACTGTGTC	144	106	NM_003048.6
*SLC9A3* (NHE3^4^)	Sodium-hydrogen exchanger 3	F: GGCAGGAGTACAAGCATCTG R: CCGCCTTCTTGTTCTGGTTG	150	95	NM_001284351.3
*ACAT1*	Acetyl-CoA acetyltransferase 1	F: CAGTATTGGGTGCAGGCTTAC R: CTGCCACCATCACATCCTGA	132	96	NM_000019.4
*HMGCS2*	3-hydroxy-3-methylglutaryl-CoA syhtnase 2	F: GAGGGCATAGATACCACCAATG R: TGGCATAACGACCATCCCA	100	105	NM_005518.4

^1^F = Forward, R = Reverse.

^2^Efficiency =  − 1 + 10^(−1/slope)^ × 100.

^3^National center for biotechnology information (NCBI; http://www.ncbi.nlm.nih.gov/).

^4^Common name.

### Statistical analysis

Data for both experiments were analyzed as a completely randomized design with a split plot treatment arrangement with a factorial in the whole plot using the MIXED procedure of SAS (version 9.4; SAS Inst. Inc., Cary, NC). For TEER and butyrate disappearance data, media treatment (VC, BCP, BUT, and BB) and time (24 and 48 h) were considered fixed effects. For cytotoxicity data, media treatment × time was not significant and therefore removed from the model. For BHB production data, media treatment and side (apical and basolateral) were considered fixed effects and data for 24 and 48 h were analyzed separately. For Experiments 1 and 2, BHB data were analyzed using log-transformed data due to heterogeneous variance. The alamarBlue cytotoxicity data were analyzed using orthogonal polynomial contrasts and a Tukey’s adjustment was used to correct multiple comparisons and data for each time point were analyzed separately.

For the real-time data, a non-parametric Kruskal–Wallis test was used for analysis of gene expression, followed by Wilcoxon rank sum test and Benjamini–Hochberg used for multiple comparisons, using R software (version 4.3.3). Data were visualized using either GraphPad (Prism) or the ggplot package of R^16^.

## Results

### Viability – AlamarBlue

**Fig. 1:**
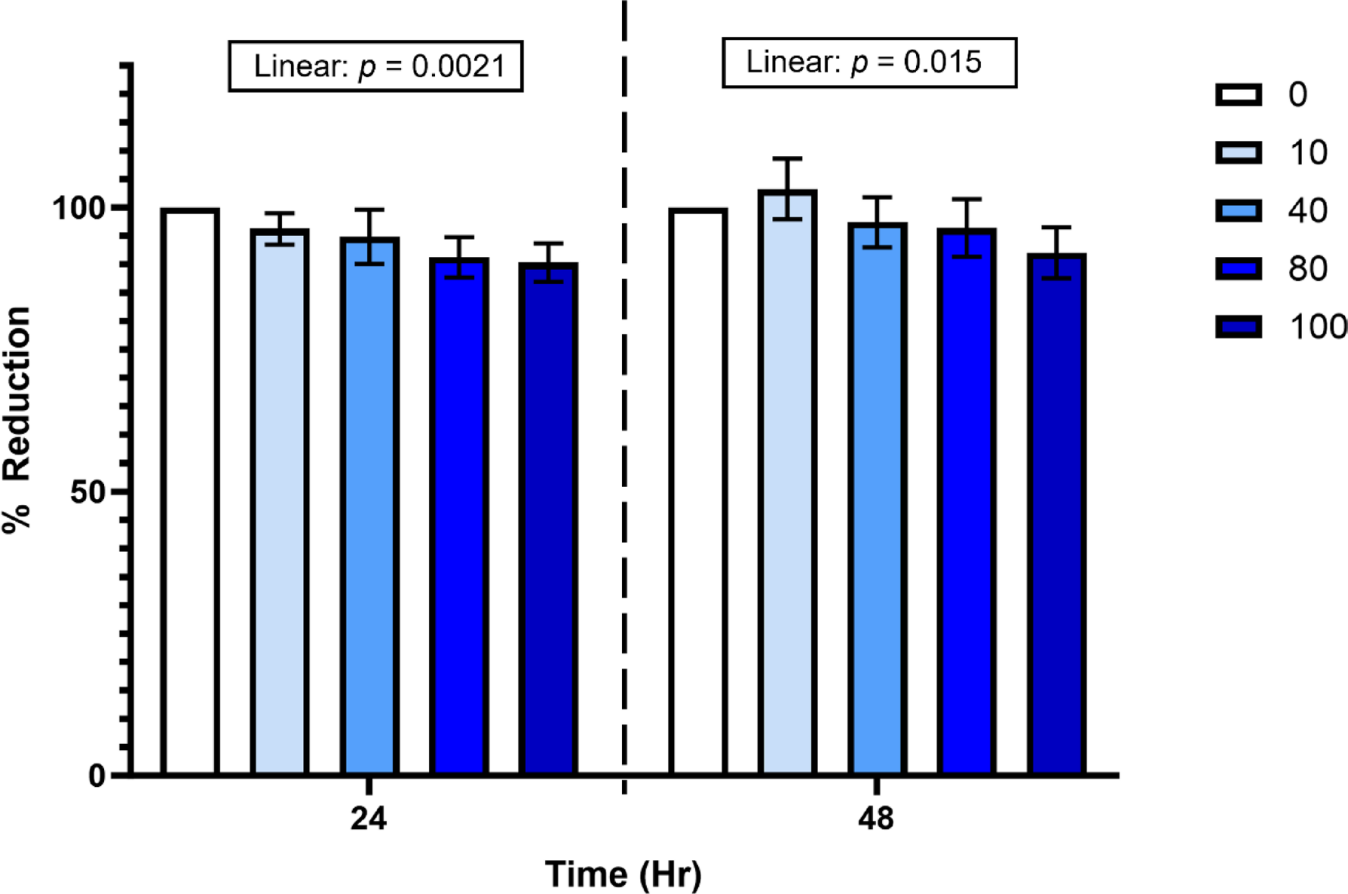
Cell viability was estimated with alamarBlue after incubation with 0, 10, 40, 80, and 100 µM beta-caryophyllene (BCP) treatment for 24 and 48 h. The treatment × time interaction was not significant and was therefore removed from the model; time was analyzed separately. Statistical significance was declared at *p* < 0.05.

Β-caryophyllene did not affect the cells at 10 or 40 µM at 24 h (Fig. 1). Although the cells were still viable, those that received 80 and 100 µM BCP exhibited a slight reduction in cellular metabolic activity compared to the cells that received 0 µM BCP (91.2 and 90.3% vs. 100 ± 3.3%, respectively; *P* = 0.048 and *P* = 0.027) at 24 h. There was no significant cellular metabolic reduction at 48 h.

### Transepithelial electrical resistance

**Fig. 2:**
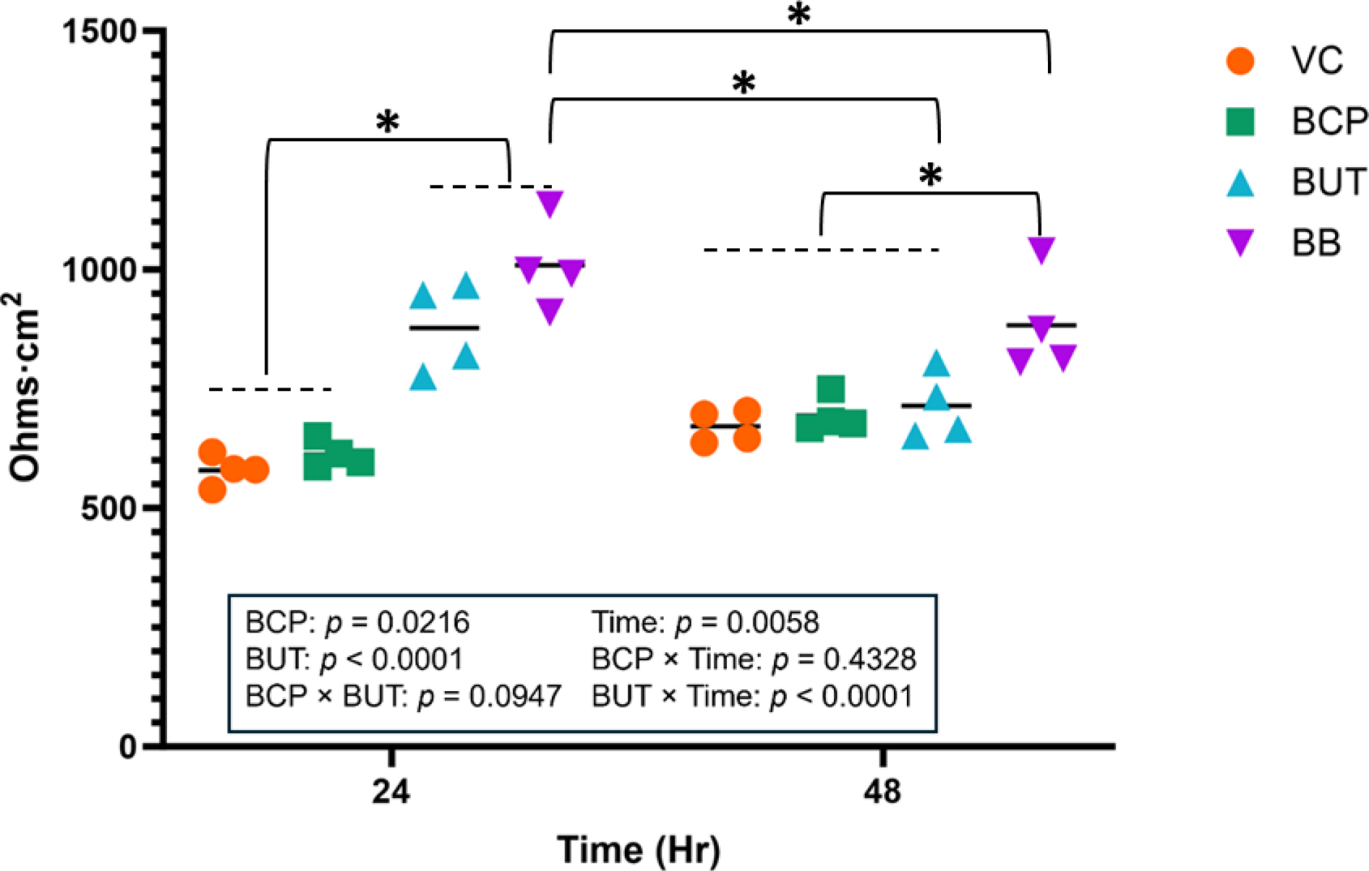
Transepithelial electrical resistance (TEER; Ohms/cm^2^) for cells (n = 4) incubated with treatments for 24 or 48 h in Experiment 1. Treatments included vehicle control (VC), β-caryophyllene (BCP), Butyrate (BUT), and Butyrate plus BCP (BB). Asterisks indicate differences between groups. Statistical significance is determined at *p* < 0.05.

In Experiment 1, both BCP (*P* < 0.05) and BUT (*P* < 0.001) increased TEER at 24 h (Fig. 2). However, only BB increased TEER at 48 h (BUT * Time *P* < 0.001).

**Fig. 3:**
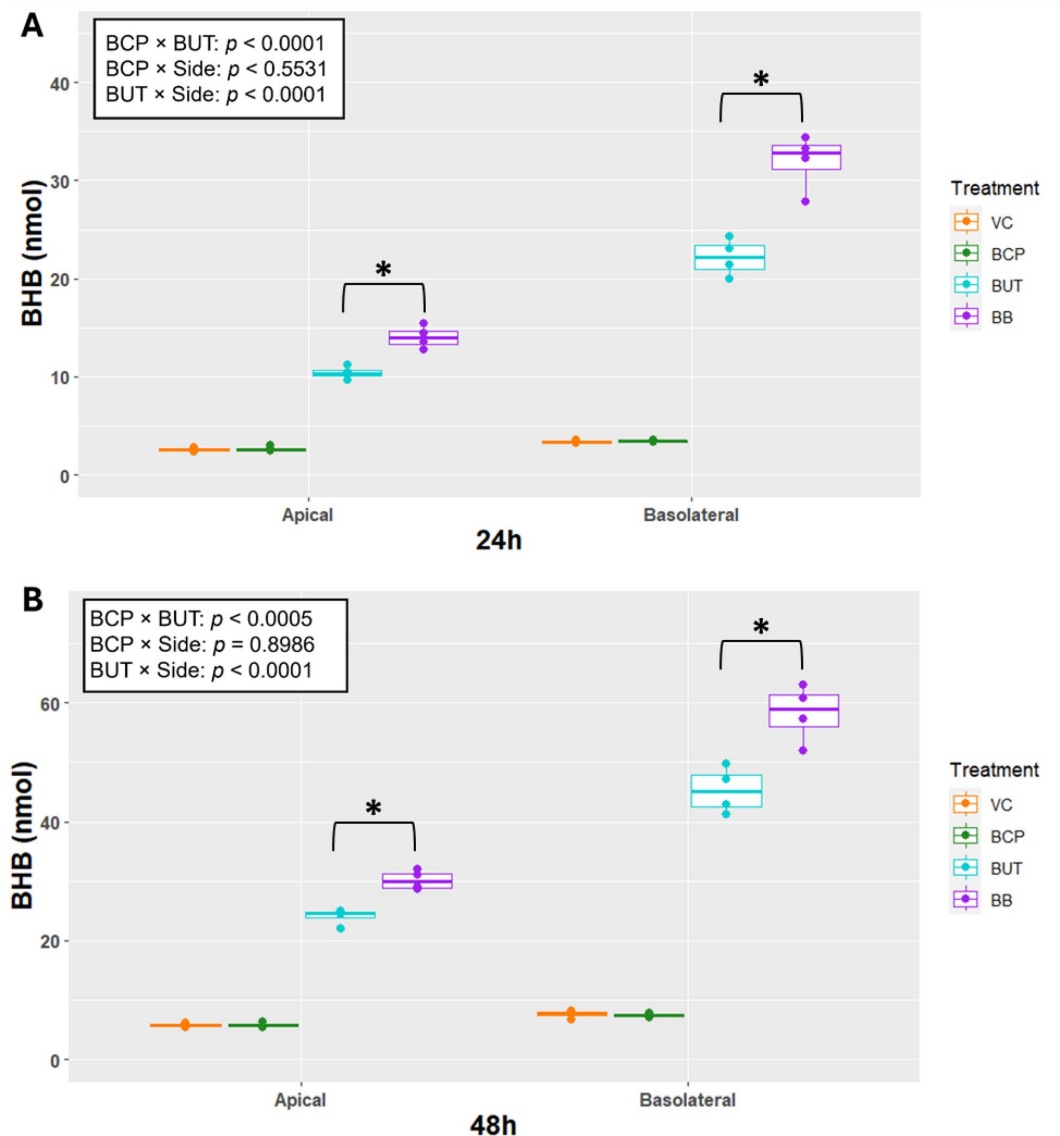
Transepithelial electrical resistance (TEER; Ohms/cm^2^) for cells (n = 5) incubated with treatments for 24 or 48 h in Experiment 2. Treatments included vehicle control (VC), β-caryophyllene (BCP), Butyrate (BUT), and Butyrate plus BCP (BB). Asterisks indicate differences between groups. Statistical significance is determined by* p* < 0.05.

In Experiment 2, both BCP (*P* < 0.05) and BUT (*P* < 0.001) increased TEER at 24 h (Fig.3 5). However, all treatments returned to baseline at 48 h (BUT * Time *P* < 0.001).

### β-hydroxybutyrate appearance and butyrate disappearance

**Fig. 4:**
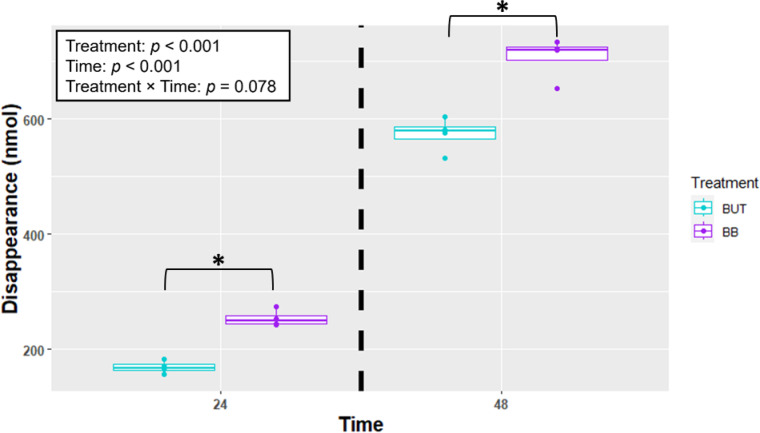
Total β-hydroxybutyrate production (BHB; nmol) for apical and basolateral side after (**A**) 24 and (**B**) 48-h incubations in Experiment 1. Treatments include vehicle control (VC), β-caryophyllene (BCP), Butyrate (BUT), and Butyrate plus BCP (BB). Statistical analysis was conducted on the log-transformed data (n = 4). Asterisks indicate differences between groups for the BCP × BUT interaction. BUT × BCP × Side was not significant and therefore removed. Data from each time were analyzed separately. Statistical significance is determined at *p* < 0.05.

For Experiment 1, production of BHB in each of the VC and BCP groups was less than 15 nmol at each time point. Regardless of time, production of BHB on the apical side exhibited a BCP × BUT interaction (*P* < 0.001) as BUT increased BHB production, but the response was greater when combined with BCP (Fig.4 3A).

 On the basolateral side, BHB production exhibited a BCP × BUT interaction (*P* < 0.001) as BUT increased BHB production, but the response was amplified when joined by BCP. Production of BHB was greater on the basolateral side by BUT (BUT * Side *P* < 0.001). This response was the same after 48 h of incubation (Fig.4 3B).

**Fig. 5:**
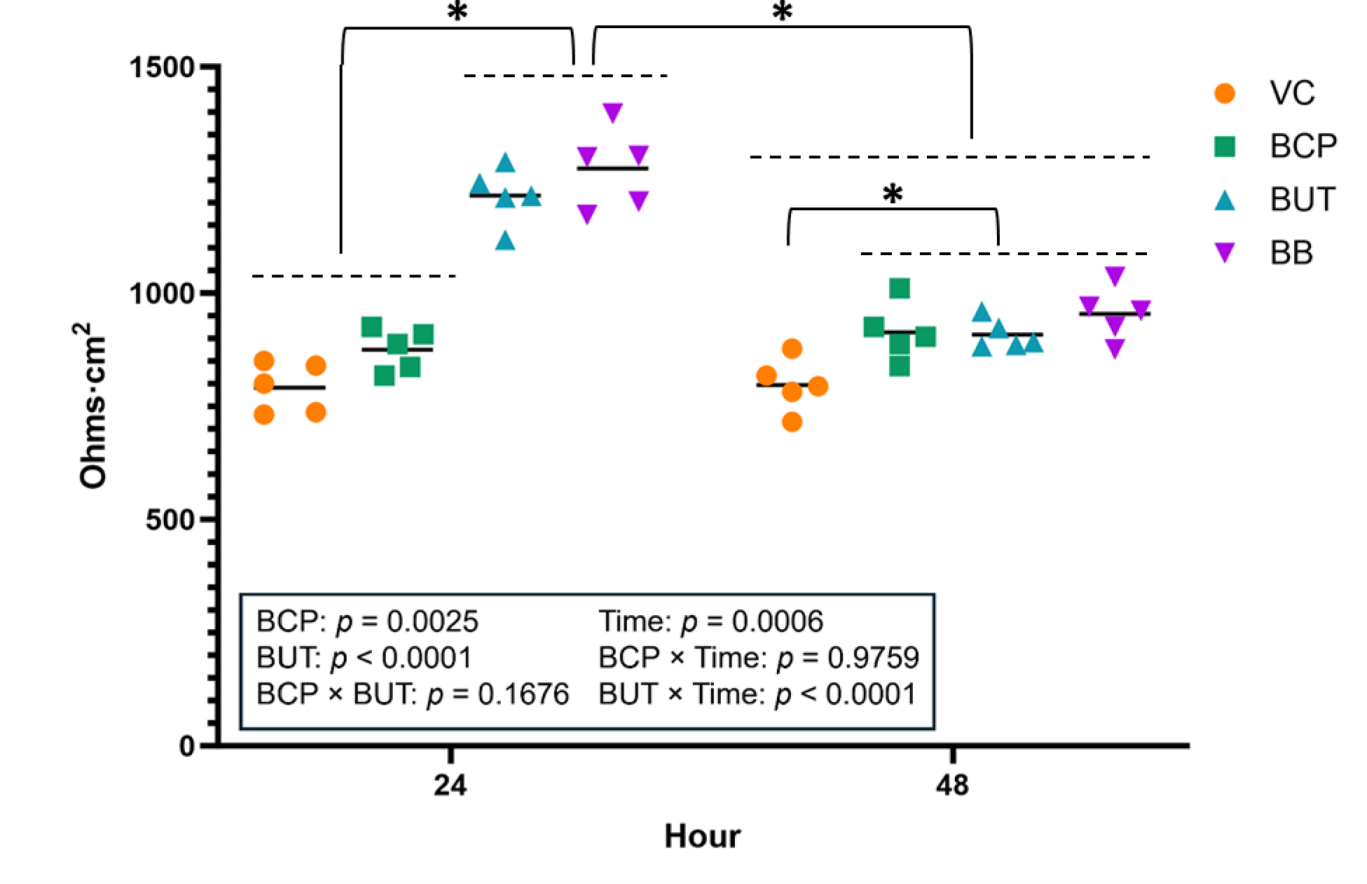
Estimate of butyrate disappearance (nmol) by cells (n = 4) incubated with treatments for 24 and 48 h in Experiment 1. Treatments include butyrate (BUT) and butyrate plus beta-caryophyllene (BB). Asterisks indicate differences between groups, within each time point. Statistical significance is determined at *p* < 0.05.

When looking at butyrate disappearance (Fig.5 4), the BB treatment had greater disappearance than BUT in 24 h (254 vs. 169 ± 12.6 nmol; *P* = 0.0005) and 48 h (707 vs. 573 ± 12.6 nmol; *P* < 0.0001) Fig. 5).

**Fig. 6:**
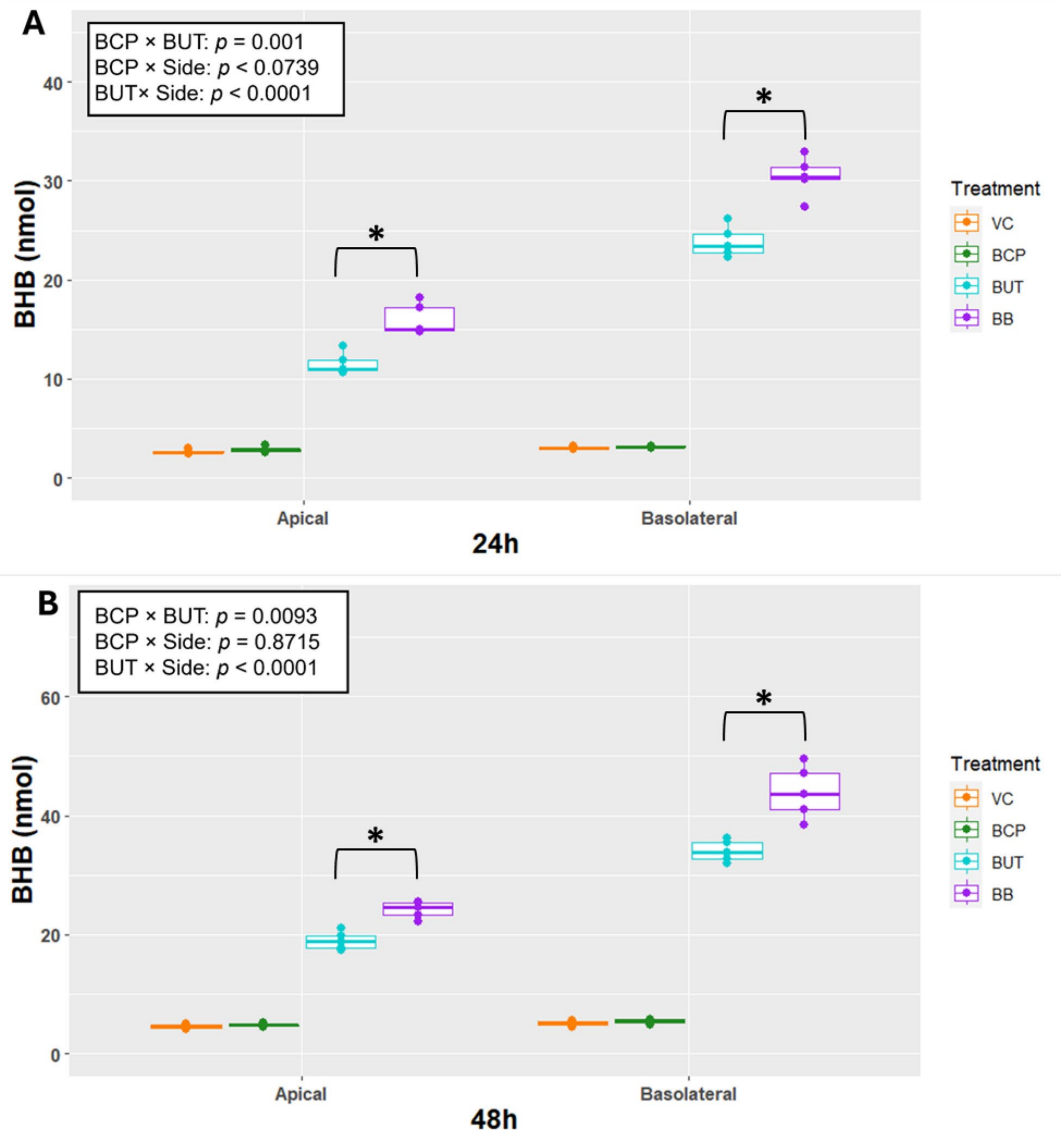
Total β-hydroxybutyrate production (BHB; nmol) for apical and basolateral side after (**A**) 24 and (**B**) 48-h incubations in Experiment 2. Vehicle control (VC), β-caryophyllene (BCP), Butyrate (BUT), and Butyrate plus BCP (BB). Statistical analysis was conducted on the log-transformed data (n = 5). Asterisks indicate differences between groups for the BCP × BUT interaction. BUT × BCP × Side was not significant and therefore removed. Data from each time were analyzed separately. Statistical significance is determined at *p* < 0.05.

For Experiment 2, production of BHB in each of the VC and BCP groups was less than 15 nmol at each time point. Regardless of time, production of BHB on the apical side exhibited a BCP × BUT interaction (*P* < 0.001) as BUT increased BHB production but the response was greater when combined with BCP (Fig. 6). Production of BHB on the basolateral side exhibited a BCP × BUT interaction (*P* < 0.001) as BUT increased BHB production but the response was greater when combined with BCP. Production of BHB was greater on the basolateral side only by BUT (BUT * Side *P* < 0.001).

**Fig. 7:**
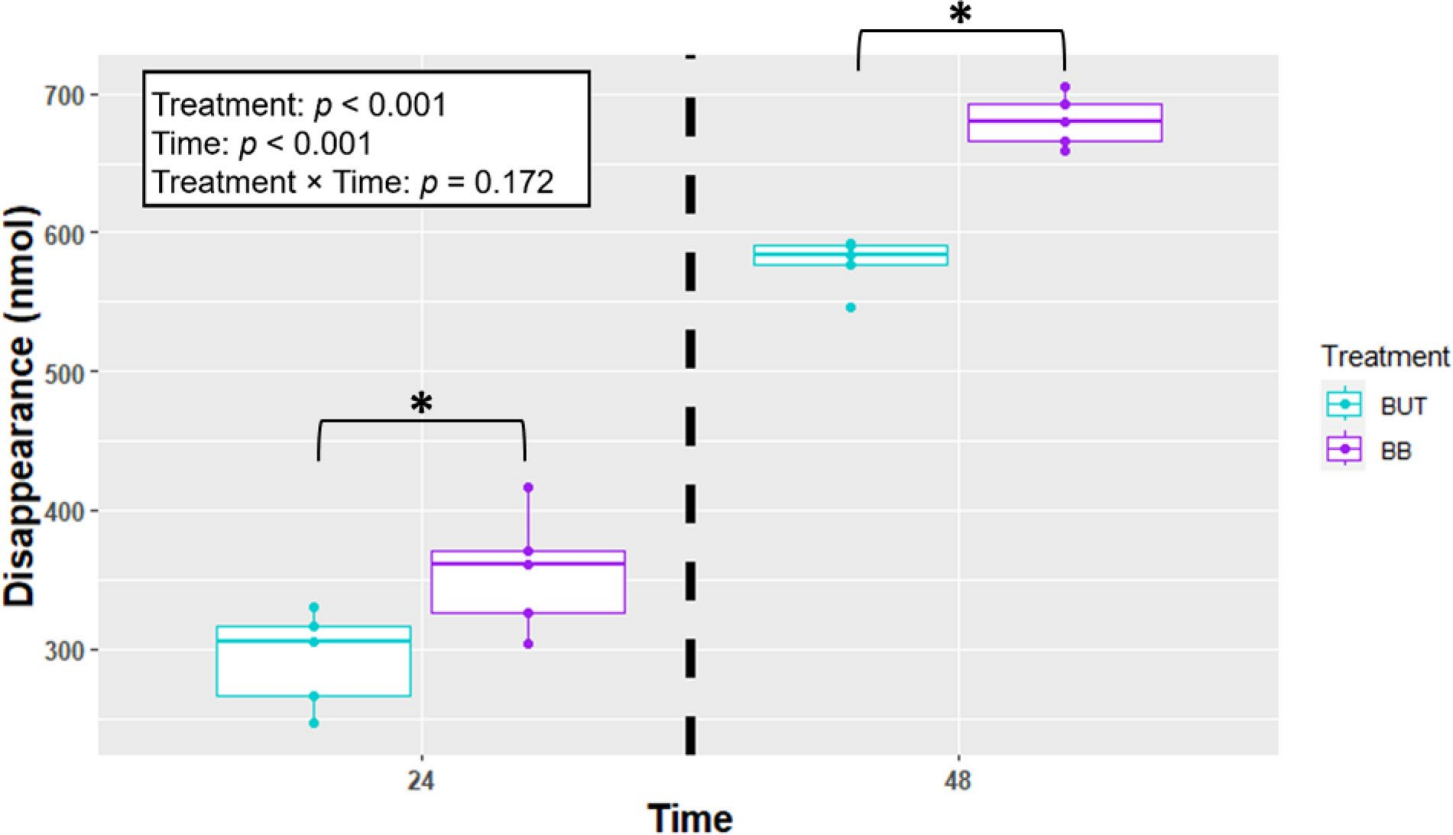
Estimate of butyrate disappearance (nmol) by cells (n = 5) incubated with treatments for 24 and 48 h in Experiment 2. Treatments include butyrate (BUT) and butyrate plus beta-caryophyllene (BB). Asterisks indicate differences between groups, within each time point. Statistical significance is determined at *p* < 0.05.

When looking at butyrate disappearance (Fig. 7), the BB treatment had a greater disappearance than BUT in 24 h (356 vs. 293 ± 13.8 nmol; *P* = 0.0053). Butyrate utilization was greater in the BB group compared to BUT after 48 h (681 vs. 578 ± 13.8 nmol; *P* < 0.0001).

### RT-qPCR gene expression

**Fig. 8:**
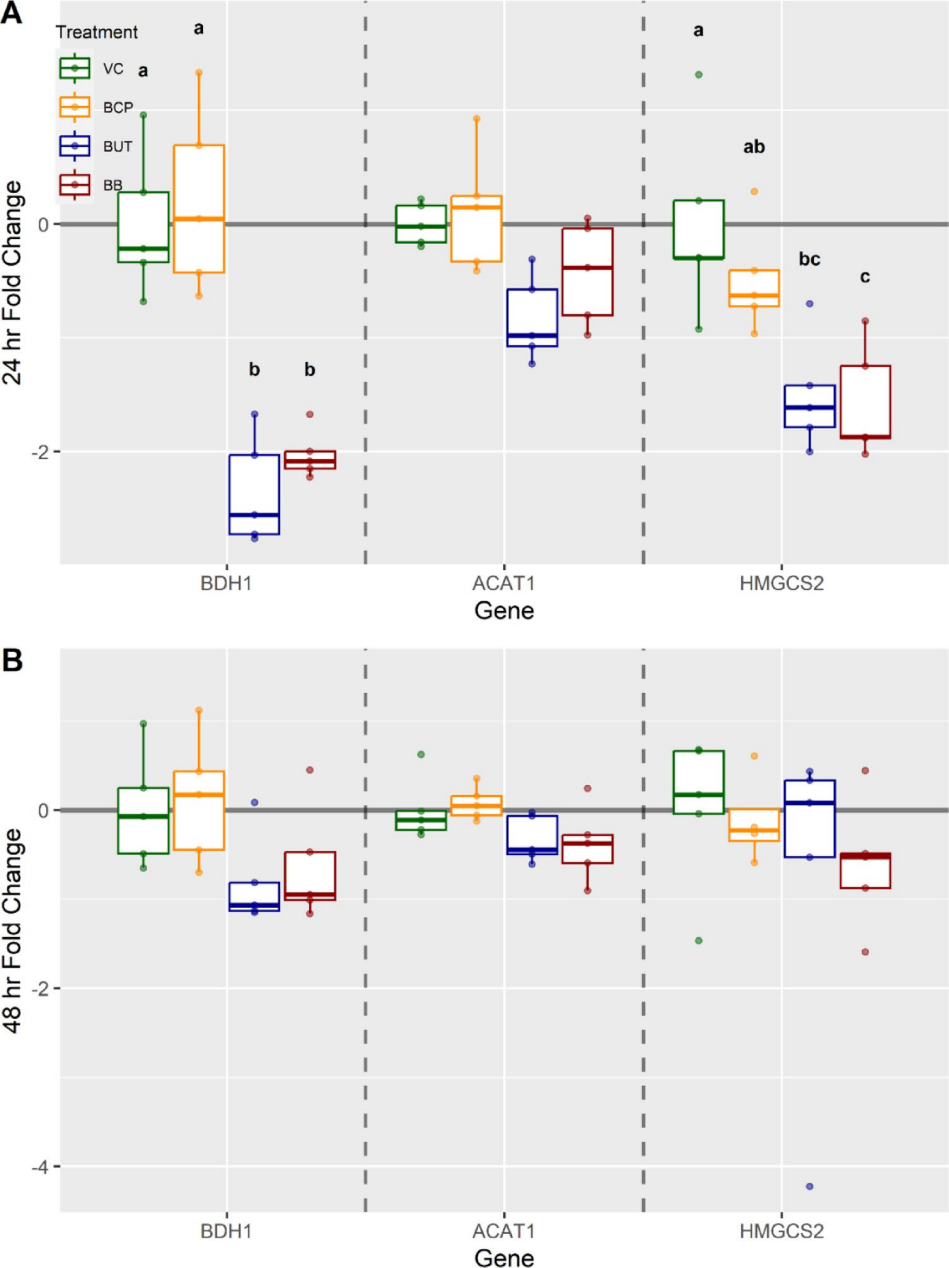
Gene expression analysis for cells (n = 5) exposed to either vehicle control (VC), β-caryophyllene (BCP), Butyrate (BUT), or Butyrate plus BCP (BB). Gene targets included: 3-hydroxybutyrate dehydrogenase 1 (BDH1), Acetyl-CoA Acetyltransferase 1 (ACAT1) and 3-hydroxy-3-methylglutaryl-CoA synthase 2 (HMGCS2) at (**A**) 24 h and (**B**) 48 h. The ΔCt was used for statistical analysis and results are presented as fold change with treatments held relative to VC. Different letters indicate significance (*p* < 0.05).

Butyrate treatment groups at 24 h expressed a 2.2-fold downregulation of *BDH1* (*P* < 0.02); at 48 h, there was no difference when compared to VC (*P* > 0.05; Fig. 8). BB groups expressed downregulation at 24 h for *BDH1* with a 1.9-fold change (*P* < 0.02; Fig. 8); however, at 48 h, there was no difference when compared to control (*P* > 0.05). BB groups expressed a downregulation of HMGCS2 by a 1.6-fold change at 24 h (*P* < 0.02; Fig. 8). BUT groups also expressed a downregulation of *HMGCS2* by a 1.6-fold change at 24 h (*P* < 0.05). There was no significant expression of *ACAT1* at either time point, regardless of treatment (*P* > 0.05; Fig. 8).

**Fig. 9:**
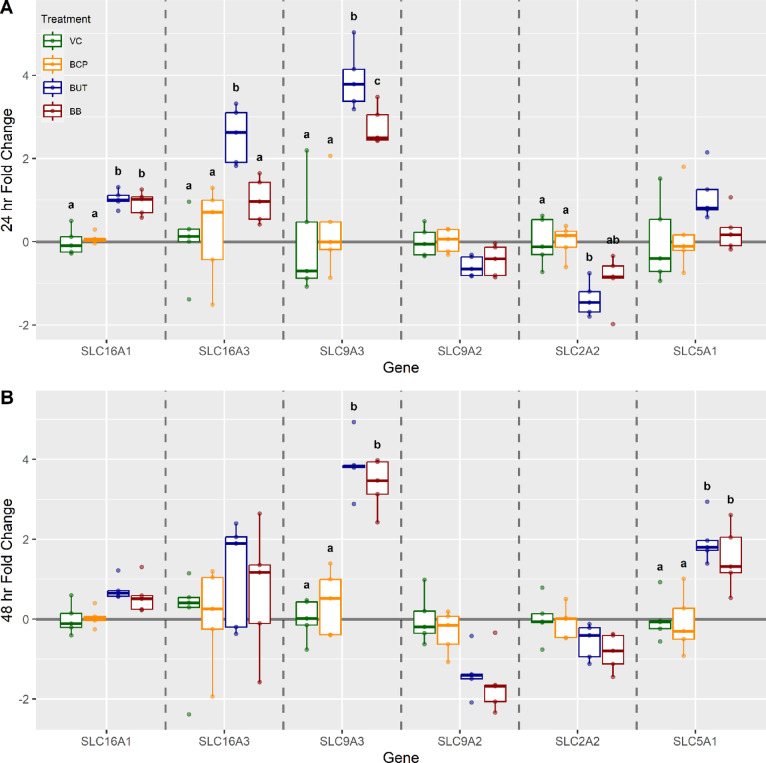
Gene expression analysis for cells (n = 5) exposed to either vehicle control (VC), β-caryophyllene (BCP), Butyrate (BUT), or Butyrate plus BCP (BB). Gene targets included: Monocarboxylate transporter 1 (SLC16A1), Monocarboxylate transporter 4 (SLC16A3), sodium-hydrogen antiporter 3 (SLC9A3), sodium-hydrogen antiporter 2 (SLC9A2), Glucose transporter 3 (SLC2A2), and Sodium-dependent glucose transporter 1 (SLC5A1) at (**A**) 24 h and (**B**) 48 h.The ΔCt was used for statistical analysis, and results are presented as fold change with treatments held relative to VC. Different letters indicate significance (*p* < 0.05).

Gene expression analysis showed that cells receiving BUT treatment alone had an upregulation in *SLC16A1, SLC16A3*, and *SLC9A3* by 1.4-, 2.5-, and fourfold, respectively (*P* < 0.05; Fig. 9A) compared to control at 24 h. There was no change in expression of *SLC5A1* at 24 h for BUT treatment (*P* > 0.05), however, at 48 h, *SLC5A1* was upregulated by a fold change of 2.1 when compared to control (*P* < 0.05; Fig. 9). Butyrate treatment groups experienced a downregulation of *SLC2A2*, with a fold change of 1.6 at 24 h (*P* < 0.05; Fig. 9) when compared to control. Cells receiving BB had an increase in *SLC16A1* expression, with a fold increase of 1.4, and *SLC9A3*, with a fold increase of 3.4, compared to control groups (*P* < 0.05; Fig. 9). There was no upregulation of *SLC5A1* at 24 h for treatment with BB (*P* > 0.05; Fig. 9); however, at 48 h, *SLC5A1* was upregulated by a fold change of 1.8 when compared to control (*P* < 0.05). Cells showed no significant expression of *SLC9A2* regardless of time or treatment (*P* > 0.05).

**Fig. 10:**
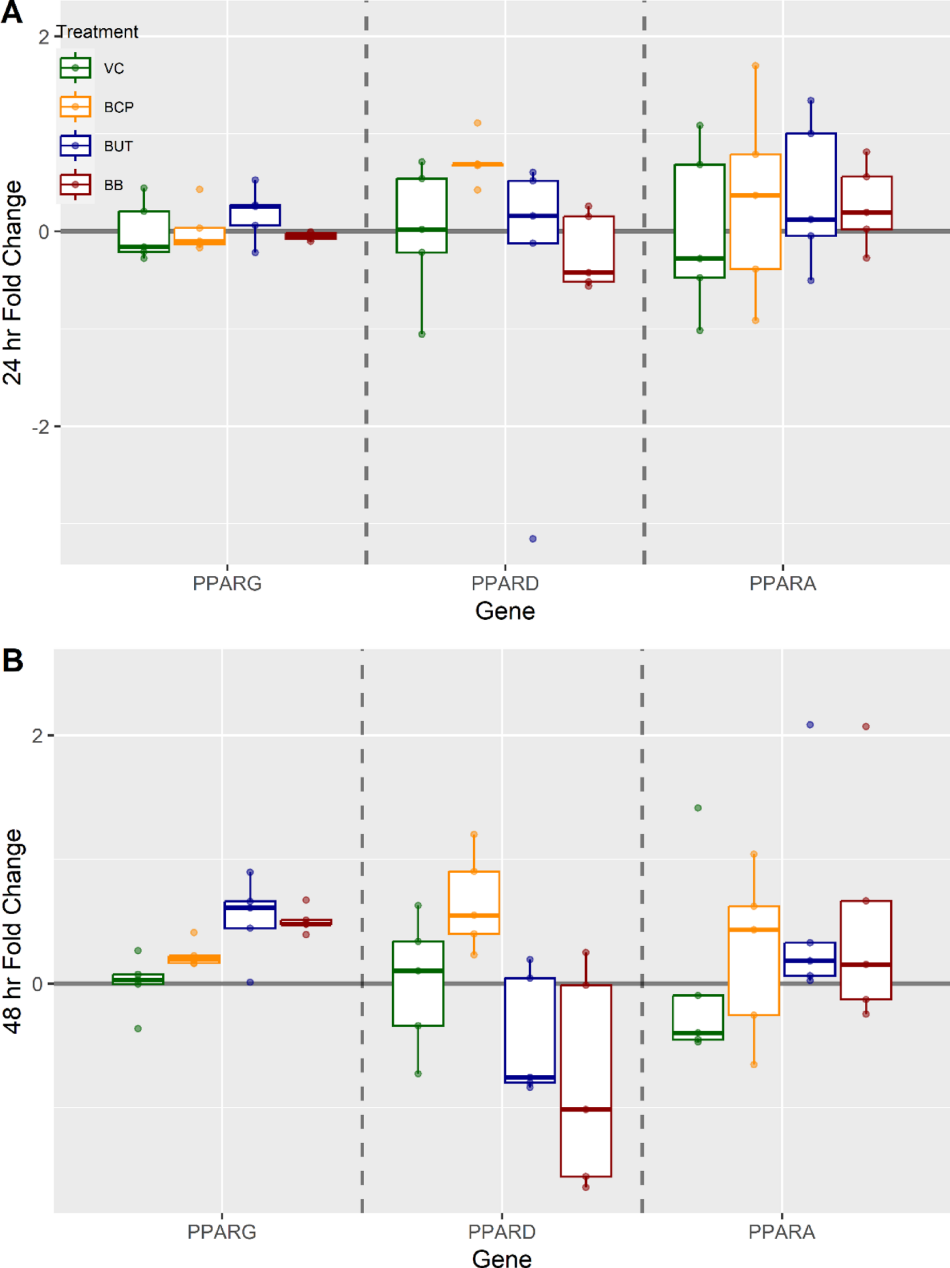
Gene expression analysis for cells (n = 5) exposed to either vehicle control (VC), β-caryophyllene (BCP), Butyrate (BUT), or Butyrate plus BCP (BB). Gene targets included: Peroxisome proliferator-activated receptor gamma (PPARG), Peroxisome proliferator-activated receptor delta (PPARD), Peroxisome proliferator-activated receptor alpha (PPARA) at (**A**) 24 h and (**B**) 48 h. The ΔCt was used for statistical analysis, and results are presented as fold change with treatments held relative to VC. Different letters indicate significance (*p* < 0.05).

There was no significant changes in gene expression of *PPARG*, *PPARD* and *PPARA* at either time point regardless of treatment (*P* > 0.05; Fig. 10).

## Discussion

Investigation into the interactions between plant-derived compounds and cellular metabolism can elucidate mechanisms of action and aid in identifying potential therapeutic targets, and development of novel alternative drugs. This knowledge could eventually be beneficial for both human medicine and livestock production. There is a deficit of studies investigating plant compounds with direct bioactivity in gastrointestinal epithelial cells, however, previous work has demonstrated that they can be used in human medicine^1^. For example, Metformin, a plant-derived compound from *Galega officinalis*, acts on epithelial cells of the GIT and is commonly prescribed to help patients living with Type II diabetes^17^. Additionally, in livestock, plant-derived compounds, such as biochanin A and thymol, have the potential to increase the overall health and performance of animals by altering microbial fermentation in the GIT^18,19^. These examples suggest that a number of plant-derived compounds have bioactivity, either through interactions with the microbial population or directly with host cells. The present study contributes to this growing body of knowledge through its investigation into the effects of BCP on butyrate utilization in intestinal epithelial cells.

While TEER can only be used to estimate barrier integrity, results showed that the addition of butyrate increased the epithelial resistance of the Caco-2 cells, suggesting that butyrate alone could improve barrier integrity. This finding is consistent with other studies where, at 2 mmol/L, butyrate increased TEER in vitro in Caco-2 cells^19^. Additional analyses, such as a permeability test or tight junction localization, are necessary to confirm the ability of BCP to improve barrier function during perturbation. In the first experiment, cells that received both BCP and butyrate had an additive effect and sustained a greater TEER for longer, compared to butyrate alone, which returned to baseline after 48 h. This additive effect was unexpected, but, may be due to resource partitioning in the cells as a result of the BCP treatment. However, in the second experiment, the combined butyrate and BCP group also returned to baseline by 48 h. While the reason for this discrepancy is unknown, these results highlight the importance of replicating experiments where appropriate. As previously discussed, BCP is shown to alter nutrient utilization, which may affect the assembly of tight junction proteins. Research suggests BCP can affect claudin-1, e.g., mice fed a high-fat diet exhibited a decrease in expression of claudin-1 and BCP d claudin-1 expression^10^. BCP may be acting upon tight junction proteins in the presence of butyrate. While this was beyond the scope of the current study, future work should explore in more depth the effects of BCP on tight junction gene expression in order to understand implications for gastrointestinal barrier function. More research is needed to investigate the effects of BCP on increasing barrier resistance, and to fully determine the therapeutic potential in human patients or animals suffering from leaky gut or colitis.

Results from the present study showed that the addition of BCP to butyrate in the cell culture media promoted greater disappearance of butyrate, and increased the production of BHB on the basolateral side. These effects were observed in both experiments, suggesting that while the effects of BCP on the TEER remains inconclusive, the effects on cellular metabolism were reliably reproducible. Butyrate can be metabolized by intestinal epithelial cells in several ways, such as conversion to BHB via ketogenesis or, oxidation via the TCA cycle. Our results suggested that, BCP affected the utilization of butyrate as an energy source and may alter resource partitioning, which is consistent with previous studies suggesting that BCP can change glucose and lipid metabolism^1^. Rodríguez-Mejía et al. showed that dosing BCP in mice resulted in less SCFA in the feces^10^. Utilization seems to change with the activation of the CB2 receptor in the ECS, as it has been shown to promote the oxidation of fatty acids through an SIRT1/PGC1α pathway^4^. Studies have suggested this increase in oxidation may be beneficial for mitigating the effects of diabetes when there is lipid dysregulation due to the reduced buildup of fat that can result in insulin resistance^4,20^. Specifically, the SIRT1 pathways work with PARRs to crosstalk with these insulin resistance diseases^21^. Not only does activation of the endocannabinoid system (ECS) result in increased expression of peroxisome proliferator receptors (*PPARG, PPARA, PPARD*), but it can also be activated during ketogenesis^22,23^. β-hydroxybutyrate metabolism involves 3-hydroxy-3-methylglutaryl-CoA synthase 2 (*HMGCS2*), Acetyl-CoA Acetyltransferase 1(*ACAT1*), and 3-hydroxybutyrate dehydrogenase 1(*BDH1*), three key enzymes that catalyze the reactions of ketogenesis^24^. Studies have shown that BCP can activate *PPARA*, which results in the upregulation of *HMGCS2*, therefore initiating the ketogenic pathway^25^. This was not seen in the present study, however, butyrate at 24 h downregulated *BDH1* and *HMGCS2*. This expression pattern could be a result of the sampling time and the current energy balance; if the cells had sufficient energy, it could have resulted in a shift away from ketogenesis. Activation of *PPARA* and *HMGCS2* could have occurred before the 24-h mark, therefore, the cells may have initiated ketogenesis prior to sample collection^26^. The considerable increase in butyrate utilization, in addition to producing BHB, could be a result of oxidation of butyrate via the TCA cycle for energy^6^. These data indicate that BCP alters energy metabolism in a cell culture model, however additional studies, including those using in vivo models are required to confirm the therapeutic potential of BCP.

B-caryophyllene may not only affects the metabolism of butyrate but also the transport. For the current study, genes of interest included monocarboxylate transporter 1(*SLC16A1* aka MCT1), which is a solute carrier that aids in transporting butyrate across the cell membrane from the apical side^27^. Monocarboxylate transporter 4 *(SLC16A3* aka MCT4*),* which is found on the basolateral side, also transports SCFA and ketone bodies^28^. Additionally, other genes that are associated with the transport of SCFA in intestinal epithelial cells, specifically sodium-hydrogen antiporters (*SLC9A2* and *SLC9A3;* NHE2 and NHE3*,* respectively), as they regulate intracellular pH by pumping out hydrogen ions and creating an influx of sodium, indirectly affecting SCFA absorption by creating an electron gradient^29^. In BUT groups, expression of *SLC16A1* (MCT1)*, SLC16A3* (MCT4), and SLC9A3 (NHE3) was upregulated, suggesting these transporters are responsible for transporting butyrate into and BHB out of the cell^27^. In this study, the addition of BCP decreased the expression of *SLC16A3* (MCT4), suggesting BCP may inhibit transport of monocarboxylates. This is consistent with other studies that have suggested some plant compounds, such as flavonoids, are weak inhibitors of MCTs^30^. Beta-caryophyllene could be affecting energy storage or BHB production since, at collection, the basolateral transporter was down-regulated. Genes related to sodium/glucose cotransporter 1 (*SLC5A1* aka SGLT1) and glucose transporter 2 (*SLC2A2* aka GLUT2) were also investigated to gain insight into how butyrate and BCP can affect glucose uptake^31^. Results from this study showed upregulation of *SLC5A1*(SGLT1) with BUT treatment, which suggests possible modification of glucose transport. This is consistent with other studies that saw upregulation of SGLT1 with dosing of sodium butyrate in mice liver cells^32^. The addition of BCP tended to decrease the expression of *SLC5A1*(SGLT1), suggesting that BCP may inhibit transport of glucose*.* After 48 h, *SLC5A1*(SGLT1) was upregulated for both BUT and BB, possibly due to decreasing concentrations of butyrate. *SLC2A2* (GLUT2) was downregulated with BUT treatment; however, this conflicts with other studies that suggest that GLUT2 is typically upregulated with butyrate supplementation^33^. This variation may be a result of the collection time, as in the mentioned study, GLUT2 expression was higher at 4 h than over 24 h. In that study, GLUT2 expression decreased as time went on, therefore, collection time can affect gene expression level. With the changes observed in glucose transporter expression, future research should include analyzing glucose levels when cells are exposed to BCP. Additionally, while involvement of the CB2 receptor could not be confirmed due to a technical issue, future work should validate its role in regulating the relevant cellular processes.

Caco-2 cells provide a very reliable model for understanding intestinal epithelial cell functions^34^, and for exploring the bioactive potential of novel compounds. However, there are some limitations. Future directions should include investigation into validating genes at the protein level, and analyses, such as a permeability test or tight junction localization, to confirm the ability of BCP to improve barrier function during perturbation. Additionally, culture of a single cell type removes the complexity of in vivo models, which may influence the effects observed. For example, there is no mucus layer, presence of bile salts, or the movement of gut contents associated with peristalsis. This model also limits the ability to look at BCPs’ effects on the gut microbiota, which may affect these results as BCP has shown to have antimicrobial activity^35^. Future work should build on the current study through the use of more complex models to fully understand the bioactivity of BCP and the potential clinical applications.

## Conclusion

Results from the current study suggest that BCP can influence multiple physiological functions in cells. The data showed that BCP may promote the utilization of butyrate by intestinal epithelial cells and increase TEER. While the physiological mechanisms involved in the observed effects need to be elucidated, our findings indicate that BCP had minimal cytotoxicity in the Caco-2 cells, while providing intriguing evidence of cellular modulation of barrier and metabolic function. Therefore, BCP is an ideal candidate for further exploration into its use as a novel and safe therapy for both humans and animals.

## Supplementary Information

Below is the link to the electronic supplementary material.

Supplementary Material 1

## Data Availability

The data used and analyzed during the current study are available from the corresponding author upon request.

## Abbreviations

BB: β-Caryophyllene and butyrate
BCP: β- Caryophyllene
BHB: β-Hydroxybutyrate
BUT: Butyrate
ECS: Endocannabinoid system
TEER: Transepithelial electrical resistance
