# Supplementary material for: The effects of β-caryophyllene on butyrate utilization and metabolism in Caco-2 cells
Source: Sci Rep. 2026 Apr 1;16:15357. doi: 10.1038/s41598-026-46790-6 (PMC13183966; doi:10.1038/s41598-026-46790-6)
Supplement: Supplementary file 1 — Supplementary Material 1 [file 41598_2026_46790_MOESM1_ESM.docx]

Supplementary Figure S1. Experimental Timeline


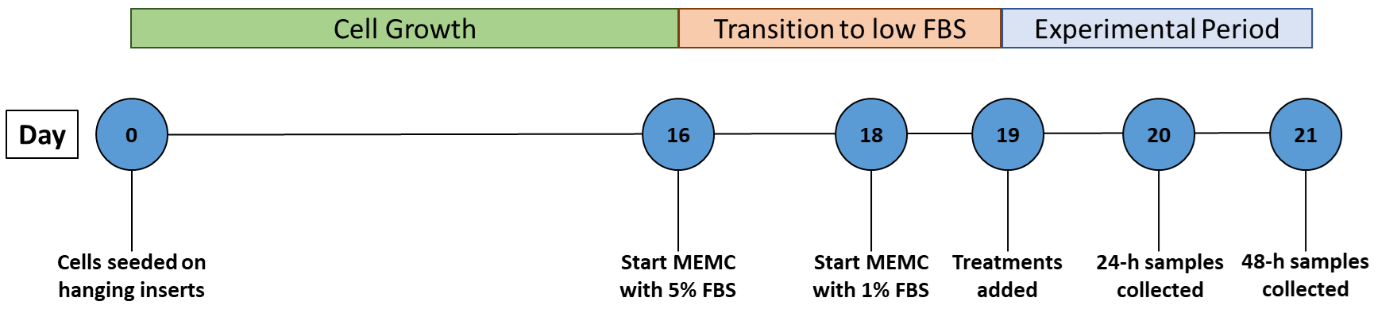


Supplementary Table S1. Liquid chromatography mobile phase gradient program.

| **Time**  **(minutes)** | **%A**  **(H2O, 0.1% formic acid)** | **%B**  **(Acetonitrile, 0.1% formic acid)** | **Flow Rate**  **(ml/min)** |
| --- | --- | --- | --- |
| 0 | 90 | 10 | 0.25 |
| 0.8 | 90 | 10 | 0.25 |
| 15 | 43 | 57 | 0.25 |
| 18 | 10 | 90 | 0.25 |
| 19 | 10 | 90 | 0.25 |
| 20 | 90 | 10 | 0.25 |
| 24 | 90 | 10 | 0.25 |

Supplementary Table S2. Mass spectrometer ion transitions and energies for compounds of interest and internal standards.

| **Analyte** | **Precursor ion**  **(m/z)** | **Quantification ion**  **(m/z)** | **Cone**  **(V)** | **Collision Energy**  **(V)** |
| --- | --- | --- | --- | --- |
| BHB | 238 | 194 | 30 | 10 |
| Int Std BHB-d4 | 242 | 194 | 30 | 8 |
| Butyrate | 222 | 152 | 36 | 12 |
| Int Std Butyrate-d7 | 229 | 153 | 36 | 14 |
